# Biological State Markers of Alcohol Abuse

**Published:** 1994

**Authors:** Mikko Salaspuro

**Affiliations:** Mikko Salaspuro, M.D., Ph.D., is the chairman of the Research Unit of Alcohol Diseases, University of Helsinki, Helsinki, Finland

## Abstract

Certain changes in blood chemistry may serve as markers of alcohol consumption. These markers can be useful in the early diagnosis, prevention, and treatment of alchol-related problems.

Biological markers are among the most promising tools for the early diagnosis and treatment of alcohol-related problems. A biological marker is generally an abnormal result of a blood test or other laboratory analysis, indicating the presence of an abnormal state or condition. This abnormal condition may refer to the presence of or susceptibility to a disease; it also may refer to an abnormal state brought about by exposure to a toxic substance, such as alcohol. Biological markers of alcohol abuse^1^ most often result from the excessive release of normal cell contents into the blood or urine. Laboratory signs of excessive and harmful alcohol consumption can be divided into two main groups: trait markers and state markers.

Trait markers are genetic, biochemical, or behavioral characteristics associated with a susceptibility to alcoholism. To be useful as trait markers, such characteristics must be detectable throughout a person’s life—not only during drinking bouts but also during abstinence and prior to the person’s first exposure to alcohol. Certain genes and proteins have been found to be more common in alcoholics and their families than in the general population and may serve as trait markers to identify people who may be susceptible to developing alcoholism. However, knowledge about the value of trait markers is still preliminary.

State markers reflect chemical changes occurring in the body as a result of alcohol use. Such markers provide no information about a person’s preexisting susceptibility to alcoholism but indicate recent or long-term alcohol consumption. They are used to detect, at an early stage, levels of alcohol consumption that are likely to be harmful if continued. This allows intervention and treatment before severe medical effects or negative social consequences associated with alcohol abuse have appeared.

Two types of state markers are used in clinical practice. The first type detects recent drinking in individuals. For example, alcohol in the urine can be measured to monitor abstinence during alcoholism treatment. The other type of biological marker is a screening tool for identifying alcoholics among the general population. Screening tests are less sensitive than monitoring tests and react only to long-term heavy drinking.

The usefulness of a marker is measured in terms of its sensitivity and specificity. Sensitivity represents a test’s ability to detect the disease in patients who have the disease. Specificity represents a test’s ability to rule out disease in patients who do not have the disease.

The ideal marker should be sensitive, specific, simple, noninvasive, and inexpensive. No currently available test fulfills all the criteria of an ideal marker (Rosman 1992; Mihas and Tavassoli 1992; Salaspuro and Roine 1994).

This article reviews the most important state markers, with special emphasis on their use in the diagnosis, prevention, and followup of alcohol-related problems and the evaluation of treatment outcome.

## Markers of Recent Alcohol Abuse

### Alcohol

Simple and specific evidence for recent alcohol use can be obtained by the detection of alcohol itself in blood, breath, urine, sweat, or saliva. Testing for alcohol has been recommended as a routine procedure when patients are admitted to emergency rooms or trauma centers (see the article by Soderstrom, pp. 127–130). Regular determination of urinary alcohol concentration also is used to assess the reliability of self-reports of alcohol consumption by patients undergoing alcoholism treatment or participating in treatment outcome studies (Orrego et al. 1979).

Detection of alcohol alone, however, does not distinguish between a single drinking episode and long-term alcohol use. The National Council on Alcoholism and Drug Dependence has suggested the following blood alcohol concentrations (BAC’s) as criteria for a preliminary diagnosis of alcoholism:^2^

- above 0.1 percent in a routine examination
- above 0.15 percent in a patient showing no signs of intoxication
- above 0.3 percent at any time.

### Acetate

The usefulness of acetate as a marker derives from its role in alcohol metabolism. Most ingested alcohol is transformed in the liver to a chemical called acetate. This transformation is initiated by the enzyme alcohol dehydrogenase (ADH). The type of chemical reaction by which alcohol becomes acetate is called oxidation.

The rate at which acetate is released from the liver to the bloodstream indicates the rate at which alcohol is oxidized. In alcoholics, the oxidation of alcohol is accelerated. This was demonstrated by results of an experiment in which 65 percent of intoxicated alcoholics were found to have elevated acetate levels compared with 8 percent of intoxicated nonalcoholics (Korri et al. 1985).

Analytical methods for the determination of acetate in the blood are simple and inexpensive. However, acetate is best suited for screening for alcoholism among intoxicated emergency room patients or alcohol-impaired drivers, because markedly elevated acetate concentrations in the blood occur only in the presence of alcohol.

### Methanol

Methanol (wood alcohol) is a naturally occurring chemical related to beverage alcohol (ethanol).^3^ Methanol is a minor ingredient of many alcoholic beverages, and some also is formed naturally in the body. Like beverage alcohol, methanol is oxidized in the liver by ADH. This enzyme oxidizes beverage alcohol in preference to methanol. Therefore, long-term ingestion of beverage alcohol inhibits the oxidation of methanol, which then accumulates in the blood. Significantly elevated levels of methanol have been found in the blood of alcoholics compared with nonalcoholics arrested for alcohol-impaired driving (Roine et al. 1989). After the beverage alcohol has disappeared from the blood, methanol is rapidly oxidized and eliminated. Thus methanol, as with acetate, is suitable only for the screening of intoxicated persons.

### 5-Hydroxytryptophol

The chemical 5-hydroxytryptophol (5–HTOL), found in urine, normally is a minor transformation product of the brain chemical serotonin. Alcohol alters the metabolism of serotonin, resulting in increased levels of 5–HTOL in the urine. As opposed to acetate and methanol, 5–HTOL can be found in urine for several hours after alcohol itself is no longer detectable. Because urinary 5–HTOL levels may be influenced strongly by common dietary factors, the marker normally is expressed as the ratio in urine of 5–HTOL to 5–hydroxyindoleacetic acid, the major transformation product of serotonin. Although this test appears useful in revealing recent alcohol intake, the analytical method is too cumbersome for routine use (Carlsson et al. 1993).

## Markers of Long-Term Alcohol Abuse

### Gamma-Glutamyl Transferase

The enzyme gamma-glutamyl transferase (GGT) is involved in protein metabolism. Long-term excessive alcohol consumption causes GGT to be released from the liver to the bloodstream. Increased GGT levels may be detected in the blood before the development of alcohol-related liver injury, making GGT a useful marker for early intervention. At present, this test is the most commonly used single laboratory marker for the detection of alcohol abuse (Salaspuro 1986).

Studies performed during the last two decades (Salaspuro 1986) demonstrate the usefulness of GGT as a marker and the variations of GGT levels in populations of long-term alcohol-abusing patients. This variation in results reflects the variety of patient populations used in different studies. For example, alcoholics hospitalized for treatment of withdrawal symptoms^4^ typically show an elevated GGT level during the first week after admission. Thus GGT is a sensitive marker for detecting active alcoholism in this population, although its use in such a well-diagnosed population seems superfluous. However, some alcoholics may have normal GGT levels, even if they have been drinking heavily for weeks or months prior to testing.

In nonalcoholics, an acute intoxicating dose of alcohol does not increase GGT levels in the blood. Neither is GGT activity influenced by drinking 60 grams of alcohol (equivalent to about five standard drinks) daily for 3 weeks (Salmela et al. 1994). Accordingly, elevated GGT most often indicates long-term heavy drinking rather than recent drinking. The probability that a patient has been consuming more than six drinks per day over a period of weeks increases from 20 percent to 50 percent when GGT exceeds 60 international units per liter of blood serum.

It has been reported (Waldstein and Skude 1979) that GGT stays within the normal limits in episodes of binge drinking of less than 2 weeks. In binges lasting 2 to 6 weeks, GGT may exceed the upper limit of normal, and binges of more than 6 weeks may increase GGT levels considerably. Patients with preexisting liver injury, however, may show an increase in serum GGT after a single oral dose of alcohol (about six standard drinks for a 160-pound person) (Nemesânszky et al. 1988).

The half-life^5^ of increased GGT is about 26 days and, consequently, in patients with very high initial GGT levels, elevated levels can be found even after an abstinence period longer than 1 month. During abstinence of only 1 week, GGT decreases in almost all alcoholics (Weill et al. 1988). Accordingly, a decrease in GGT during total abstinence lasting not more than 7 days indicates an “alcoholic” origin of elevated GGT with a sensitivity of 90 percent.

The primary disadvantage of GGT as a screening test of alcohol abuse is its poor specificity in population samples containing mostly nonalcoholics. In a random sample of middle-age Swedish men (age 48) (Kirstenson et al. 1980), 16 percent had elevated GGT. In 75 percent of the subjects, the main reason was excessive alcohol consumption. Other significant causes of elevated GGT included various medications for epilepsy, inflammation, or pain and factors related to diet, such as being more than 30 percent overweight or having elevated levels of fatty substances in the blood. In populations with lower prevalence of excessive alcohol consumption, however, only 50 percent of elevated GGT levels may be due to alcohol (Penn and Worthington 1983).

Other nonalcoholic origins of elevated GGT levels include some heart and kidney diseases, severe trauma, and all types of liver disease. Medications known to increase GGT levels include barbiturates as well as medications used to treat epilepsy and blood-clotting disorders. In patients using these medications, a slightly elevated GGT level has no diagnostic importance.

### Mean Corpuscular Volume

Macrocytosis (increased size of red blood cells) is a marker of excessive alcohol consumption, expressed as the average size of the red blood cells in a blood sample (mean corpuscular volume, or MCV). The mechanism by which alcohol increases MCV is unknown but may involve direct toxicity of alcohol to immature red blood cells in the bone marrow. In various patient populations, increased MCV is found in 26 to 94 percent of alcoholic patients. Increased MCV correlates positively with the duration of the drinking episode and the total amount of alcohol consumed during the episode (Tönnesen et al. 1986). Because of the long life of red blood cells, MCV values return to normal slowly (within months) during abstinence.

As a biological marker of alcohol abuse, increased MCV is less sensitive, but more specific, than GGT. The incidence of macrocytosis among patients in general practice has been reported to be 2.4 percent (Seppä et al. 1991). Of those patients with macrocytosis, 80.2 percent of men and 34.1 percent of women were alcohol abusers. Other causes of increased MCV include deficiencies of nutrients (e.g., vitamin B_12_ and folate), liver disease, reticulocytosis (an increase in the number of immature red blood cells), antiepileptic drugs, increased age, smoking, and menopause (Seppä et al. 1991).

MCV can be measured inexpensively in most hospital laboratories. However, MCV is probably of greater value in estimating the duration and extent of actual drinking episodes in heavy-drinking patients than in screening for alcoholism (Tönnesen et al. 1986).

### Carbohydrate-Deficient Transferrin

Transferrin is a blood protein that functions in the transport of iron through the bloodstream. Carbohydrate-deficient transferrin (CDT) is an abnormal variant of transferrin found in increased amounts in persons abusing alcohol (Stibler 1991). The sensitivity of CDT levels as a marker for alcoholism ranges from 9 percent to greater than 90 percent. Sensitivity may be lowest among patients with significant liver damage. Compared with other biological markers of alcohol abuse, the major advantage of CDT is its high specificity, with a type of non-alcohol-related cirrhosis being the only disease producing high CDT values (in approximately 30 percent of these cases.) Rare genetic conditions also influence CDT levels (Stibler et al. 1988, 1991).

Another advantage of elevated CDT as a marker is its significantly long half-life (approximately 2 weeks). In addition, in patients with alcoholic liver disease, CDT is a better marker of abstinence than GGT or MCV because of its better sensitivity and because CDT levels are not affected by the liver injury itself.

Despite these advantages, studies on the screening of heavy drinkers from general populations indicate that the sensitivity of CDT to detect early alcohol problems may be limited. Among 77 nonalcoholic heavy drinkers, the sensitivity of CDT was 28.6 percent and specificity was 91.8 percent (Sillanaukee et al. 1993). In the same study, the best conventional marker, GGT, correctly detected 35.1 percent of heavy drinkers with a specificity of 86.9 percent. Unsatisfactorily low sensitivities in the detection of heavy drinking have been reported also in other screening studies focused on normal populations, alcohol-impaired drivers, young problem drinkers, and university students (Nilssen et al. 1992; Nyström et al. 1992).

## Marker Combinations

Several investigators have tried to combine two or more different laboratory tests to achieve better sensitivity and specificity in screening for alcohol abuse. For example, a combination of seven different laboratory tests reflecting blood abnormality, liver injury, the effect of alcohol on fat metabolism, and tolerance to alcohol has been shown to detect alcohol abuse with a sensitivity of 82 percent in men and 71 percent in women, whereas the level of the “best” single test (GGT) was increased in only 49 percent of these patients (Cushman et al. 1984).

Combining an indicator of liver injury (GGT) with a blood marker (MCV) increased the sensitivity from 78 percent and 72 percent for each test respectively, to 92 percent for both together. However, the specificity of this combination was only 40 percent (Stamm et al. 1984).

Findings from our experiments show that the combination of acetate and GGT correctly detects 71 percent of heavy drinkers, while the percentage for GGT alone was only 35 percent. Thus, the combination of several markers may increase the sensitivity to detect alcohol abuse but may do this by compromising specificity and increasing cost (Korri et al. 1985).

Sensitivity also can be improved by applying a statistical procedure known as discriminant function analysis to combinations of biological markers. Using this method with MCV, GGT, and the enzyme alkaline phosphatase (a marker of non-alcohol-related liver injury), it was possible to detect correctly more than 80 percent of patients with excessive alcohol consumption in a hospital study of 512 patients (Chalmers et al. 1981).

The basis of the discrimination is that the heavy drinkers tend to have elevated MCV and GGT but only a slightly raised level of alkaline phosphatase. A discriminant function analysis based on multiple biochemical analysis and some physical findings was shown to have a sensitivity of 78 percent in the detection of high alcohol use among 201 males in a community sample (Vanclay et al. 1991). Discriminant function analysis of blood test results could be a potentially useful tool in the detection of alcohol abuse. However, it is not widely available and might be too expensive for everyday clinical use, especially if the number of parameters to be measured is too large.

## Markers in Clinical Practice

Markers have been used successfully in the early detection of heavy drinking. This was demonstrated by a large Swedish study in which middle-age men were screened for alcohol abuse using GGT.

Those with increased GGT activity were divided into intervention and nonintervention groups and followed for 6 years. The intervention group received counseling that focused on living habits, with a goal of moderate drinking. The nonintervention group received no counseling. The results of this study show clearly that even minimal intervention is efficient in significantly reducing alcohol-related sickness and mortality (Kirstenson et al. 1983). Similar positive results also have been reported from other intervention studies (Anderson 1990). (For more information on intervention, see the article by Buchsbaum, pp. 140–145.)

The early detection of alcohol abuse and subsequent intervention should be especially encouraged in inpatient and emergency units of general hospitals (Persson and Magnusson 1989; Anderson 1990). Useful markers for this purpose include alcohol concentration in breath, blood, or urine and GGT or CDT levels in blood. Results of these tests should be confirmed with any of several standard alcohol use and alcoholism questionnaires (see the sidebar by Nilssen and Cone, pp. 136–139). Other relatively simple markers suitable for intoxicated patients include acetate and methanol concentrations in the blood.

Markers are useful during alcoholism treatment. Because of the potential unreliability of self-reports concerning drinking behavior, such markers may be the earliest and sometimes the only indicators of relapse. The determination of alcohol in blood, breath, or urine is simple and reliable for this purpose, and other conventional state markers (i.e., GGT, MCV, and CDT) may be useful as well (Behrens et al. 1988). Recovering alcoholics also can use state markers to monitor their progress during treatment.

Biological markers can improve the reliability of studies focusing on alcoholism treatment effectiveness by helping to determine correctly whether individual subjects have become abstinent (Irwin et al. 1988; Chick et al. 1988; Keso and Salaspuro 1990).

## Summary

Several laboratory abnormalities are associated with excessive alcohol consumption. These abnormalities are used as biological markers in the early detection of excessive alcohol consumption in everyday clinical practice. Such markers also are valuable in the secondary prevention of alcohol-related problems, because they can be used as motivating factors to help patients to cut down their drinking. The use of biological markers in the follow-up and evaluation of treatment results should be encouraged.

No current test can satisfy all the criteria of an ideal marker: sensitivity, specificity, simplicity, and low cost. Potential markers are being evaluated, however, and may eventually come into use.

## Footnotes

1 Throughout this article, terms such as “alcohol abuse,” “alcoholism,” “problem drinking,” and “heavy drinking” are used. There is overlap among these terms in the alcoholism literature; therefore, the wording in each case is based on the terms used in the reference cited.

2 A BAC of 0.1 percent or greater is the legal definition of intoxication in most States.

3 Throughout this article, the unmodified term “alcohol” refers to beverage alcohol.

4 A range of withdrawal symptoms may appear upon cessation of a drinking bout, including anxiety, tremors, hallucinations, and convulsions.

5 Half-life is the time required for biological activity to eliminate from the body half the quantity of a substance taken into the body.
